# Human *Toxoplasma gondii* Seroprevalence in the Canary Islands: Implications for One Health Surveillance and Control

**DOI:** 10.3390/microorganisms14010067

**Published:** 2025-12-28

**Authors:** Eligia González-Rodríguez, José Alberto Montoya-Alonso, Kevin M. Santana-Hernández, Elena Carretón, Myriam R. Ventura, Eligia Rodríguez-Ponce

**Affiliations:** 1 Department of Animal Pathology and Production, Bromatology and Food Science and Technology, University of Las Palmas de Gran Canaria, 35017 Las Palmas de Gran Canaria, Spain; eligia.gonzalez102@alu.ulpgc.es (E.G.-R.); myriam.rodriguezventura@ulpgc.es (M.R.V.); eligia.rodriguezponce@ulpgc.es (E.R.-P.); 2 Internal Medicine, Faculty of Veterinary Medicine, Research Institute of Biomedical and Health Sciences (IUIBS), University of Las Palmas de Gran Canaria, 35413 Las Palmas de Gran Canaria, Spain; alberto.montoya@ulpgc.es

**Keywords:** Canary Islands, foodborne infection, human epidemiology, toxoplasmosis, zoonoses

## Abstract

*Toxoplasma gondii* is a globally significant foodborne parasite, yet epidemiological data in Spain are limited. This study provides the first comprehensive assessment of human *T. gondii* seroprevalence across the Canary Islands and identifies key risk factors. A total of 1223 serum samples were tested for anti-*T. gondii* IgG antibodies using ELISA. Demographic and geographic data were recorded. Overall seroprevalence was 32.3%, with no significant differences between sexes. Geographic variation was notable: the highest seroprevalence occurred in the western islands (La Palma 52.4%) and the lowest in the eastern islands (Gran Canaria 17.6%). Seropositivity increased progressively with age, ranging from 0% in children (0–15 years) to 51.3% in individuals over 60 years. Climatic factors also influenced exposure, with the temperate cold isoclimate associated with higher seroprevalence and arid zones showing more seronegative cases. Logistic regression confirmed age and isoclimate as significant predictors of seropositivity. The observed decline in prevalence compared to historical data suggests improvements in hygiene, dietary practices, and public health measures. Nevertheless, moderate-to-high risk persists, particularly among older adults and in favourable environmental conditions. These findings underscore the importance for One Health strategies, i.e., food safety and education, feral cat control and human and animal surveillance.

## 1. Introduction

The Food and Agriculture Organization of the United Nations (FAO) and the World Health Organization (WHO) have identified toxoplasmosis (*Toxoplasma gondii)* as a foodborne parasitic infection of global concern [1]. It remains one of the leading causes of foodborne illness in several countries [2,3,4].

Despite the global significance of this disease, epidemiological studies in Spain remain scarce and limited in scope [5]. In Gran Canaria, a previous study conducted in 1995 by Rodríguez-Ponce et al. [6] reported an alarmingly high seroprevalence in humans (63.35%) and goats (63.31%). Later, a more recent study published in 2017 revealed a marked decrease in goat seroprevalence on the island (9.8%) as well as an overall seroprevalence of 7.8% in goats across the archipelago [7]. Although Carranza-Rodríguez et al. [8] provided updated seroprevalence data in the Canary Islands, their study included only 324 HIV-positive residents. Thus, representative data for the general population across the archipelago remain scarce, highlighting the need for broader surveys.

In humans, contact with cat feces represents an important source of infection, since cats defecate in soil and sand, and contact with these substrates constitutes a risk factor for transmission. Infection may also occur through the ingestion of contaminated milk or water, or through the consumption of poorly washed fruits and vegetables [9,10]. Consumption of undercooked meat is another major risk factor [11,12]. In the Canary Islands, the control of feral cat populations currently poses a challenge, not only for the preservation of biodiversity and endemic species but also for public health [13]. Among other measures, sterilization–and-release programmes have been implemented in different areas of the archipelago to control these populations, although no official parasite control protocols currently exist for such colonies [14].

Tourism has long been the main driver of the Canary Islands’ economy, accounting for approximately 35% of the regional GDP and around 40% of local employment. Between 2016 and 2019, the archipelago ranked among the leading European destinations, surpassing Paris, Catalonia, the Balearic Islands, and London in the number of overnight tourist stays. With a resident population of about 2.2 million, the islands received more than 15 million visitors in 2019 [15,16]. In highly touristic and densely populated areas, frequent interactions occur between people and feral cat colonies, which are often fed by tourists [17]. Direct contact between tourists and cats is not considered a risk factor for infection, but these interactions can hinder the control of stray cat populations and increase opportunities for human exposure to zoonotic pathogens. Therefore, in regions such as the Canary Islands, where tourism is a major economic sector and contact with potential reservoirs of *T. gondii* is relatively common, toxoplasmosis may represent a relevant public health consideration, particularly for immunocompromised visitors [18].

Given that no updated data are available on the seroprevalence of this pathogen in humans on the island of Gran Canaria, and that no serological study has ever been conducted across the entire Canary archipelago, the current epidemiological status of human toxoplasmosis remains unknown. Therefore, the aim of this study was to update and determine the epidemiological situation of this pathogen in the human population of the Canary Islands, including the assessment of its distribution across the different climatic regions of the archipelago.

## 2. Materials and Methods

### 2.1. Climatology of the Archipelago

To map the different climate types across the Canary archipelago, the Köppen climate classification, adapted for this region in previous research, was applied to the various municipalities [6,19]. Based on this classification, two main climatological zones were defined: the coastal area (dry zones) and the central and highland areas (temperate zones). More specifically, four distinct isoclimatic zones were identified, ascending in altitude from the coast to the central peaks of the islands: dry desert isoclimate (Dd), dry steppe isoclimate (Ds), temperate mild isoclimate (Tm), and temperate cold isoclimate (Tc).

The dry zones, located below 200 m above sea level on or near the coast, comprise two isoclimates: (i) the dry desert (Dd) isoclimate, characterized by precipitation below the archipelago’s annual average, an average annual temperature >18 °C, and very dry summers; and (ii) the dry steppe (Ds) isoclimate, also with average annual temperatures > 18 °C, a steppe-like climate, and dry summers. This dry climate predominates on the islands of Lanzarote and Fuerteventura.

The temperate zones, also known as mesothermic, have winter temperatures < 18 °C and significant rainfall during the coldest months. These zones include (i) the temperate mild (Tm) isoclimate, located between 200 and 800 m altitude, characterized by hot, dry summers and mild winters; and (ii) the temperate cold (Tc) isoclimate, found above 800 m, similar to Tm but with summer temperatures < 22 °C and colder winters. This temperate climate predominates in El Hierro, La Gomera, and La Palma.

On Tenerife and Gran Canaria, all four isoclimatic zones are present and more widely distributed across the islands.

### 2.2. Serum Samples

A cross-sectional study was conducted using a random sample of 1223 serum samples collected between March 2014 and October 2015 from residents of the seven Canary Islands. These samples were obtained from several diagnostic laboratories and had been used in a previous study [20]. The samples were originally obtained for routine checkups or due to suspected illness. Serum samples were stored at −80 °C until analysis. Information recorded for each sample included age, sex, and area of residence (city, town).

The distribution by age and sex was comparable to the population structure of the Canary Islands, according to the 2015 census data [21]. Patient confidentiality was strictly maintained, and written informed consent was obtained from all participants.

### 2.3. Serological Methods

The presence of *Toxoplasma gondii* IgG antibodies in human serum was determined using an enzyme-linked immunosorbent assay (ELISA) with microtiter plates coated with inactivated *T. gondii* antigens. All reagents were supplied by DIESSE Diagnostica Senese (Siena, Italy), and the assay was performed according to the manufacturer’s instructions.

The qualitative assessment was based on the ratio between the optical density (absorbance) of the sample and the cut-off value (calibrator 1). Samples were classified as positive if the ratio was >1.3, doubtful if between 0.7 and 1.3, and negative if <0.7.

In addition, the ELISA provides quantitative IgG concentrations (IU/mL), which were obtained using a calibration curve constructed from positive sera of known concentration. These quantitative values were not used for statistical comparisons, but only to support sample classification according to the manufacturer’s interpretative criteria. Each 96-well plate included a calibration series consisting of eight wells: three blanks (0 IU/mL) and five calibrators at 8, 20, 50, 100, and 200 IU/mL. The IU/mL values for tested samples were interpolated from this calibration curve.

Based on these criteria, samples were categorized as seropositive (anti-*T. gondii* > 10 IU/mL), seronegative (<6 IU/mL), or doubtful (6–10 IU/mL). For statistical analysis, doubtful cases—whose values were very close to the negative ratio threshold—were included in the seronegative group.

### 2.4. Data Processing and Statistical Analysis

Data were analyzed using SPSS Base 27.0 software for Windows (SPSS Inc./IBM, Chicago, IL, USA). Descriptive analyses were performed for the variables of interest, with proportions calculated for qualitative variables. The binary variable “Qualitative Seroprevalence” was defined by assigning a value of 1 to samples classified as positive according to the qualitative criteria of the commercial kit, and a value of 0 to samples classified as negative or doubtful. Proportions were compared using the chi-square test, with statistical significance set at *p* < 0.05.

A logistic regression model [22] was applied to assess the variance in seropositivity and to predict the probability of being seropositive according to the age of the participants.

The mathematical expression of the model is represented in Equation (1):

(1) p/q = e^(α + β⋅X), X ≡ “Age (years)”

where p is the probability of being seropositive, and q is the probability of the opposite event, i.e., being seronegative. The model was used to evaluate the age-related trend in seropositivity across the study population.

## 3. Results

A total of 1223 serum samples were tested, although 26 had incomplete data. The distribution by island and by age group for each island is shown in Table 1.

Overall, 387/1197 samples (32.3%) tested positive for *T. gondii* antibodies. Of these, 57.4% (n = 222) were from men and 42.6% (n = 165) from women. While the overall prevalence appeared higher in men, statistical comparisons using Fisher’s exact test for age-stratified groups with small counts (<5) and chi-square tests for larger groups revealed no significant differences between sexes in most categories.

Participants ranged in age from 2 to 91 years; for comparative purposes, samples were grouped into five age categories. Subgroup analyses by age and island showed that certain groups (e.g., 46–60 years in Tenerife and Gran Canaria) had significant sex-specific differences (*p* < 0.01).

Seroprevalence also varied across islands and age groups. Analyses using chi-square tests for larger sample sizes and Fisher exact test for smaller ones indicated significant differences in seropositivity between islands within some age categories, particularly in smaller islands such as El Hierro and La Gomera, where sample sizes were limited. These results suggest that local demographic patterns and island-specific factors may influence exposure risk to *T. gondii*. The distribution of seropositivity by island, age group, and sex is summarized in Table 2.

By island, the highest seroprevalence values were observed in the western islands—La Palma (52.4%, 98/187), El Hierro (43.7%, 35/80), Tenerife (34.5%, 98/284), and La Gomera (34.4%, 64/186)—whereas the lowest rates occurred in the eastern islands—Gran Canaria (17.6%, 19/108), Lanzarote (21.2%, 49/231), and Fuerteventura (26.7%, 65/243).

**Figure 1:**
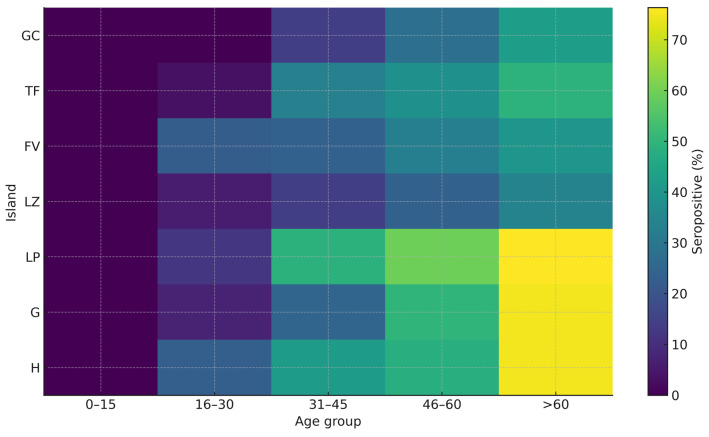
Heat map showing the percentage of seropositive samples by island and age group. Legend: GC (Gran Canaria), TF (Tenerife), FV (Fuerteventura), LZ (Lanzarote), LP (La Palma), G (La Gomera), H (El Hierro).

When analyzed by age group (Figure 1), the proportion of seropositive individuals increased significantly with age. In the 0–15 years group, all samples were seronegative (ratio = 0). In the 16–30 years group, the positive-to-negative ratio was 0.13 (seroprevalence: 11.2%); although the χ^2^ statistic could be calculated, no significant inter-island differences were detected. In the 31–45 years group, the ratio rose to 0.39 (seroprevalence: 28.2%), showing clear statistical significance, with negative sera predominating across most islands. In the 46–60 years group, the ratio reached 0.61 (seroprevalence: 37.8%), again with negative sera predominating on most islands.

A pronounced increase was observed in individuals >60 years old, with a ratio of 1.05 (seroprevalence: 51.3%), indicating a generalized rise in seropositivity. Notably, La Palma showed the highest prevalence in this age group (76.3%), compared with 45.3% in the remaining islands. These differences were statistically significant (χ^2^ = 11.34, *p* = 0.001), confirming that individuals >60 years old on La Palma had a significantly higher probability of being seropositive. Overall, these findings demonstrate a clear age-related increase in *T. gondii* seroprevalence.

This strong association with age prompted the use of a logistic regression model to predict the probability of seropositivity as a function of age. The model explained nearly 70% of the variance in the data. According to the Wald statistic [23], *p*-values < 0.05 indicated that the model provided a good fit for predicting the probability of *T. gondii* seropositivity based on age.

Regarding climatological factors, higher seroprevalence rates were found in temperate climates (Tc 46.7%; Tm 36.2%), and lower rates in drier climates (Dd 24.9%; Ds 35.3%). Statistically significant association was observed between seroprevalence and isoclimate type. Significantly more seronegative individuals were found in dry desert zones (*p* < 0.05), whereas seropositive cases were more frequent in temperate cold zones (*p* < 0.05) compared with the other isoclimatic categories of the archipelago. Due to the lack of precise location data for participants from El Hierro and La Gomera, isoclimatic assignment was not possible for these islands; therefore, a global analysis was performed based on the available data.

## 4. Discussion

According to a recent systematic review conducted in Spain, the overall seroprevalence of *T. gondii* in the general population (based on several studies between 1993 and 2023) was approximately 32.3% (95% CI: 28.7–36.2%), which is consistent with the results obtained in the present study [5]. Although the result is slightly lower than that reported by Carranza Rodríguez et al. in the Canary Islands [8], the latter was based on a limited number of samples and focused on a specific subpopulation. Therefore, it can be stated that the seroprevalence observed in the Canary Islands falls within the national average. Moreover, the seroprevalence found in the Canary Islands lies within the range of global estimates for Europe, where average values have been reported around 30–32% in the general population [24]. This result suggests that exposure to *T. gondii* in the archipelago is comparable to the European average, although lower than in eastern and western regions of the continent, where prevalence may reach up to 50%. However, it exceeds the values reported in northern Europe, where prevalence is approximately 18% [25]. The similarity with the European mean, despite the climatic and geographical particularities of the archipelago, may be explained by persistent risk factors such as the consumption of undercooked meat and environmental exposure to oocysts, combined with the influence of a temperate climate that may favour oocyst survival. These findings highlight the need to maintain control measures and strengthen public health education, particularly in a territory with high tourist influx and environmental conditions conducive to transmission.

The decline in *T. gondii* antibody prevalence over recent decades has been highlighted in numerous studies [18,26]. In the Canary Islands, the only previous data available came from one of the seven islands studied, Gran Canaria. Evidence indicates a marked decrease, as that earlier study reported a seroprevalence of 63.35% [6]. This notable reduction is likely related to increased promotion of healthy hygienic habits among residents and possibly supported by feral cat control campaigns implemented in recent decades to protect both public health and island biodiversity [27,28]. Numerous studies conducted in domestic and wild animal species have confirmed that *T. gondii* infection is widely distributed throughout Spain [29,30,31], particularly among feral cats, which represent a major source of environmental oocyst contamination [32,33]. This reduction is consistent with similar trends observed in epidemiological studies across several European countries [26,34,35].

*T. gondii* is endemic in the Canary Islands [36], and its oocysts survive better in warm and humid areas than in cold and arid regions, as well as at lower altitudes [18]. The archipelago is characterized by mild thermal conditions, minimal seasonal contrasts, and remarkable climatic variability over short distances—a phenomenon known as microclimatic diversity. This unique environmental heterogeneity has led to the classification of some of the larger islands (Gran Canaria and Tenerife) as “microcontinents,” making them ideal natural laboratories for epidemiological studies of certain diseases [37].

These environmental characteristics that favour parasite survival are consistent with the results obtained, since significantly more seronegative individuals were found in the dry desert isoclimate (arid areas), characterized by high mean annual temperatures and very dry summers, which hinder oocyst persistence in the soil [33]. Conversely, higher seropositivity was observed in the temperate cool isoclimate (Tc), where lower summer temperatures, cooler winters, and greater humidity may promote oocyst survival [38,39]. Among all the islands, La Palma has the largest area under this temperate isoclimate, which could explain the results observed in this study.

These cooler zones also correspond to more rural areas; several studies and reviews have demonstrated that residence in rural settings is associated with higher risk or seroprevalence of *T. gondii* infection. This is usually attributed to greater contact with soil and animals, consumption of locally produced (and sometimes undercooked) meat, exposure to untreated water, and lower socioeconomic and sanitary conditions [40,41].

The results show an age-related increase in seroprevalence, consistent with previous findings [8,42]. This pattern aligns with the biology of *T. gondii* infection, as exposure to the parasite typically occurs cumulatively throughout life through repeated contact with contaminated environmental or food sources. Accordingly, the likelihood of seroconversion increases progressively with age. This trend has been reported in various European populations [25,40] as well as in countries with different levels of development. Furthermore, the higher seropositivity observed in older age groups may reflect less favourable hygienic and sanitary conditions in past decades, when food safety measures and awareness of zoonotic diseases were more limited. In contrast, younger generations have grown up with improved access to treated water, refrigeration, and health education campaigns, which may explain their lower exposure rates. From an epidemiological standpoint, the positive association between age and seroprevalence supports the hypothesis of a continuous but relatively low-intensity transmission pattern over time, rather than sporadic outbreak dynamics. Since *T. gondii* infection is usually subclinical and immunity is long-lasting, cumulative seropositivity reflects historical exposure of the population [43]. In this context, seroepidemiological analysis remains a valuable indirect tool to assess environmental risk patterns and changes in lifestyle and dietary habits over time.

Control measures aimed at reducing the risk of toxoplasmosis as a food- or waterborne infection [44] include restricting stray cat access, implementing effective rodent control programmes, thoroughly washing fruits and vegetables, and freezing meat before consumption. Culinary tourism, increasingly popular as part of travel experiences, may complicate the application of such preventive measures. Travellers consume food prepared outside the home and thus have limited control over food handling and hygiene practices [45]. Additionally, tourists often believe that feeding feral cats benefits animal welfare, increasing close contact between cats and humans and, consequently, the risk of zoonotic transmission [46]. Therefore, raising awareness about potential foodborne risks and providing appropriate information to reduce infection risk in endemic areas are essential [18].

Importantly, the findings of this study also provide concrete evidence supporting a One Health perspective, as they highlight the close interconnection between environmental factors, animal reservoirs, and human practices in shaping *T. gondii* transmission in the archipelago. The association between seroprevalence and specific isoclimates reflects how land characteristics—particularly humidity and moderate temperatures—favour oocyst survival in soil and water. These microclimatic patterns interact with land-use practices in rural and peri-urban areas, where agricultural activity, livestock presence, and frequent soil contact create ecological niches that sustain the transmission cycle [6].

Feral and free-roaming cats, identified as key reservoirs and environmental contaminators, are especially abundant in areas with predictable food sources and limited population control, reinforcing their central role in environmental contamination [13,14]. Human behaviours, such as the consumption of locally produced meat, inadequate washing of vegetables, or feeding of feral cats—particularly frequent in tourist zones—further modulate exposure risk [33,46].

In this context, integrated environmental and land-management strategies, including protection of water sources, monitoring contamination in high-risk microhabitats, and cat population control programmes, should be combined with public health education and veterinary surveillance. The interplay between climate, ecological reservoir hosts, and human practices demonstrated in this study underscores the need for multisectoral One Health policies to mitigate *T. gondii* transmission in the Canary Islands.

This study provides the first comprehensive seroepidemiological data on human toxoplasmosis in the Canary Islands. *T. gondii* infection is a complex disease influenced by multiple environmental and demographic factors. Further research is needed to identify specific environmental risk factors for transmission [47]. Moreover, Sepúlveda-Arias et al. [18] noted that non-immune travellers from areas with low *Toxoplasma* prevalence who visit high-prevalence regions may be at increased risk of infection. Hence, serological screening can help assess population health status and guide prophylactic strategies. In this regard, the One Health concept is crucial for preventing zoonotic diseases in both animals and humans, particularly considering their close interconnection.

As a limitation, although univariate analyses were performed in the present study to describe the distribution of seroprevalence across age groups, sexes, and islands, this approach does not account for potential interactions between variables. Multivariate analyses could provide further insights into the relative contribution and interplay of risk factors, such as age, sex, and geographic location. Future studies with larger sample sizes and additional covariates are warranted to explore these interactions in more detail.

## 5. Conclusions

The results of this study reveal a favourable epidemiological shift in *Toxoplasma gondii* seroprevalence among the population of the Canary Islands, with a notable decline compared to previous decades in Gran Canaria. This reduction suggests improvements in sanitary conditions and in the population’s hygiene and dietary habits. However, the overall prevalence remains high, indicating a persistent moderate-to-high risk that warrants continued epidemiological surveillance.

Age and isoclimate were identified as key risk factors associated with *T. gondii* exposure. Seroprevalence increased progressively across older age groups, while the humid and temperate climatic zones of the archipelago appeared to favour oocyst survival and, consequently, parasite transmission. Although cats—particularly feral ones—play a central role in the biological cycle of *T. gondii*, the main infection routes remain indirect, involving the consumption of contaminated food or water and contact with contaminated soil.

Population mobility, tourism, and migratory flows may influence the future epidemiology of toxoplasmosis in the region, increasing exposure risk for non-immune individuals. Therefore, this study underscores the need for integrated policies under the One Health approach, encompassing joint human–animal health surveillance, cat population management, educational programmes, and food safety measures to reduce infection risk and protect public health.

## Figures and Tables

**Table 1:** Distribution of serum samples by age and island. n: total sample size. %: Percentual representation of each island in the total amount of sera. n: sample size used for statistics. SE: standard error. Min.: Minimum. Max.: Maximum.

Island/Sex	n	%	Missing Data	N	Mean Age ± SE	Min. Age	Max. Age
Gran Canaria (GC)	115	9.0	7	108	39.58 ± 1.59	4	75
Tenerife (TF)	293	23.9	7	286	47.60 ± 1.02	2	91
Fuerteventura (FV)	249	20.1	8	241	41.95 ± 0.087	2	73
Lanzarote (LZ)	232	19.3	1	231	48.88 ± 0.87	12	86
La Palma (LP)	187	15.6	-	187	47.35 ± 1.11	8	80
La Gomera (G)	64	5.4	-	64	42.98 ± 1.65	10	83
El Hierro (H)	83	6.7	3	80	43.68 ± 1.62	2	82
Total	1223	100%	26	1197	45.43 ± 0.044	2	91

**Table 2:** Distribution of serum samples by Age Groups, Island and Sex (F: Female; M: Male). N: sample size. T: total by age Group. %: global percentage by age group.

Age Group	0–15		16–30		31–45		46–60		>60		Total	
Island/sex	F	M	F	M	F	M	F	M	F	M	F	M
Gran Canaria (GC)	5	7	7	8	29	13	12	13	5	9	58	50
Tenerife (TF)	3	3	19	11	60	45	43	33	44	25	169	117
Fuerteventura (FV)	6	4	8	23	47	63	26	41	12	11	99	142
Lanzarote (LZ)	0	2	4	12	30	41	37	64	18	23	89	142
La Palma (LP)	0	3	13	12	30	27	40	24	19	19	102	85
La Gomera (G)	0	1	1	12	9	11	9	17	2	2	21	43
El Hierro (H)	0	1	1	12	8	23	10	17	3	5	22	58
N	14	21	53	90	213	223	177	209	103	94	560	637
T (%)	35 (2.9)		143 (11.9)		436 (36.4)		386 (32.2)		197 (16.6)		1197	

## Data Availability

The original contributions presented in this study are included in the article. Further inquiries can be directed to the corresponding authors.
